# CRISPR-targeted genome editing of mesenchymal stem cell-derived therapies for type 1 diabetes: a path to clinical success?

**DOI:** 10.1186/s13287-017-0511-8

**Published:** 2017-03-09

**Authors:** Dario Gerace, Rosetta Martiniello-Wilks, Najah Therese Nassif, Sara Lal, Raymond Steptoe, Ann Margaret Simpson

**Affiliations:** 1 The School of Life Sciences, Chronic Disease Solutions Team and the Centre for Health Technologies, University of Technology Sydney, PO Box 123, Broadway, NSW 2007 Australia; 2 Translational Cancer Research Group, University of Technology Sydney, Sydney, Australia; 3 Neuroscience Research Unit, University of Technology Sydney, Sydney, Australia; 4 The University of Queensland Diamantina Institute, The University of Queensland, Translational Research Institute, Brisbane, Australia

**Keywords:** Mesenchymal stem cells, Type 1 diabetes, Clinical trials, Immunomodulation, Insulin-producing cells

## Abstract

Due to their ease of isolation, differentiation capabilities, and immunomodulatory properties, the therapeutic potential of mesenchymal stem cells (MSCs) has been assessed in numerous pre-clinical and clinical settings. Currently, whole pancreas or islet transplantation is the only cure for people with type 1 diabetes (T1D) and, due to the autoimmune nature of the disease, MSCs have been utilised either natively or transdifferentiated into insulin-producing cells (IPCs) as an alternative treatment. However, the initial success in pre-clinical animal models has not translated into successful clinical outcomes. Thus, this review will summarise the current state of MSC-derived therapies for the treatment of T1D in both the pre-clinical and clinical setting, in particular their use as an immunomodulatory therapy and targets for the generation of IPCs via gene modification. In this review, we highlight the limitations of current clinical trials of MSCs for the treatment of T1D, and suggest the novel clustered regularly interspaced short palindromic repeat (CRISPR) gene-editing technology and improved clinical trial design as strategies to translate pre-clinical success to the clinical setting.

## Background

Type 1 diabetes (T1D) results from an organ-specific autoimmune-mediated loss of insulin-secreting β cells in the pancreas. People with T1D manage their blood glucose levels using exogenous insulin therapy; however, this does not eliminate the development of long-term diabetic complications such as retinopathy, nephropathy, and neuropathy [1]. In addition, life-long use of exogenous insulin increases the risk of hypoglycaemic unawareness which can be potentially life-threatening.

Currently, pancreas or islet transplantation remains the only cure; however, these treatments are limited by a shortage of donor organs and the requirement for life-long immunosuppression [2]. An alternative to current therapies has been the generation of surrogate β cells from a variety of tissue sources via chemically or genetically induced transdifferentiation [3–10]. However, many surrogate β-cell solutions that are derived from cells such as induced pluripotent [7] and embryonic stem cells [11] are susceptible to allorejection or recurrent autoimmunity. Thus, long-term survival of transplanted cells requires encapsulation which does not necessarily provide immune protection in all cases. Due to their high plasticity, immunomodulatory properties, and fewer ethical concerns, mesenchymal stem cells (MSCs) are an attractive alternative target cell for the autologous and allogeneic treatment of T1D without the requirement for encapsulation. In addition, early studies demonstrating the ability of MSCs to differentiate into insulin-producing cells (IPCs) via ex vivo chemical induction [6–10] or various gene therapy approaches [12, 13] qualifies them as ideal candidates for cell transplantation.

The purpose of this review is to highlight the success of MSC-derived therapies in pre-clinical models of diabetes and reflect on the failure of the translation of these studies into the clinical setting. We therefore suggest the utilisation of clustered regularly interspaced short palindromic repeat (CRISPR) and implementing improved clinical trial design for the future success of T1D MSC-derived therapies.

### MSCs as an immunomodulatory therapy for T1D

Within the context of T1D, MSCs have been assessed for use as an immunomodulatory therapy in small animal models of diabetes such as non-obese diabetic (NOD) mice [14–16] and streptozotocin (STZ)-induced diabetic animals [17, 18], where improvements in the glycaemic control of treated animals have been observed. Through these early studies, MSC interventions demonstrated improved T1D outcomes through two mechanisms: (i) MSC migration to areas of pancreatic injury and modification of the islet microenvironment to promote the survival and regeneration of surviving β cells; and (ii) abrogating inherent autoimmunity against β cells [19]. In addition, MSCs can be co-transplanted with pancreatic islets, thereby protecting the islets from allogeneic immune responses [20]. The success of MSC infusions in pre-clinical animal models of diabetes are summarised in Table 1.

**Table 1:** Summary of the major MSC-derived studies in pre-clinical animal models of T1D

Treatment type	Intervention	Outcomes	Fresh/frozen	Reference
Immunomodulation	Mice received 1 × 10^6^ AD-MSCs by i.p. injection	Reversal of hyperglycaemia characterised by increased serum insulin, amylin, and GLP-1 levels. Downregulation of the CD4^+^ Th1-based immune response and expansion of T_regs_ in the pancreatic lymph nodes.	Fresh	[13]
	Mice received 1 × 10^5^ MSCs either i.p. or i.v.	Reduced infiltration of T cells to pancreatic islets associated with preferential migration of MSCs to pancreatic lymph nodes.	Fresh	[14]
	Mice received 0.5 × 10^6^ MSCs administered systemically	Reduced blood glucose levels and an increase in morphologically normal pancreatic islets.	Fresh	[16]
	Rats received 2–4 × 10^6^ MSCs via tail vein injection	Enhanced insulin secretion and sustained normoglycaemia. Islets from treated rats co-expressed high levels of *Pdx-1* and insulin.	Fresh	[17]
	Mice received a co-transplantation of primary hBMSCs and human islets at serial ratios under the kidney capsule	Good blood glucose control and increased levels of serum insulin and C-peptide when islets were co-transplanted with hBMSCs. hBMSCs also increased the percentage of T_regs_ and prevented cytokine-induced loss-of-function of transplanted islets.	Fresh	[19]
	Mice received 5 × 10^5^ MSCs injected i.v. once a week for 4 weeks	BALB/c-MSC trafficked to the pancreatic lymph nodes of treated animals. Administration of BALB/c-MSC temporarily resulted in reversal of hyperglycaemia in 90% of treated animals.	Fresh	[30]
IPC differentiation	Chemical differentiation	BMSCs formed islet-like clusters containing IPCs that expressed multiple pancreatic genes. The clusters released insulin in a glucose-dependent manner and ameliorated diabetes in STZ-treated nude mice.	Fresh	[6]
	Chemical differentiation	BMSCs differentiated into IPCs and acquired islet-like architecture after transplantation, developed an endocrine gene expression profile and demonstrated glucose-responsive insulin secretion. Subcapsular renal transplantation of these aggregates lowered circulating blood glucose levels.	Fresh	[8]
	Chemical differentiation	Differentiated BMSCs expressed multiple pancreatic genes and exhibited glucose-responsive insulin secretion. Transplantation into STZ-diabetic mice imparted reversal of hyperglycaemia and an improved IPGTT.	Fresh	[9]
	Chemical differentiation	Differentiation cells expressed pancreatic genes and displayed glucose-responsive insulin secretion. Transplantation of differentiated cells into diabetic rats reduced blood sugar levels.	Fresh	[33]
	Viral-mediated differentiation	Differentiated cells expressed all four islet hormones and demonstrated glucose-responsive insulin secretion. Cell transplantation into STZ-diabetic immune-deficient mice resulted in further differentiation, including induction of *NeuroD1* and reduction of hyperglycaemia.	Fresh	[11]
	Viral-mediated differentiation	hMSCs differentiated into IPCs that expressed multiple islet genes and released insulin/C-peptide in a weak glucose-responsive manner. Upon transplantation into STZ-diabetic mice, normoglycaemia was obtained within 2 weeks and maintained for at least 42 days.	Fresh	[12]
	Viral-mediated differentiation	Differentiated AD-MSC expressed some islet genes and secreted increasing amounts of insulin in response to increasing concentrations of glucose. Transplantation in STZ-diabetic rats resulted in lowered blood glucose and higher glucose tolerance.	Fresh	[36]
	Viral-mediated differentiation	Expression of *Pdx1* in AD-MSCs did not induce the pancreatic phenotype in vitro. Upon transplantation, the cells engrafted in the pancreas, wherein they expressed insulin and C-peptide, significantly decreased blood glucose levels, and increased survival.	Fresh	[37]
	Viral-mediated differentiation	Body weight in diabetic mice that received GFP-mMSCs expressing the human insulin gene was increased by 6% within 6 weeks after treatment.	Fresh	[39]

*AD-MSC* adipose-derived mesenchymal stem cell, *BMSC* bone marrow mesenchymal stem cell, *CD* cluster of differentiation, *GFP* green fluorescent protein, *GLP-1* glucagon-like peptide 1, *hBMSC* human bone marrow mesenchymal stem cell, *i.p.* intraperitoneal, *IPC* insulin producing cell, *IPGTT* intraperitoneal glucose tolerance test, *i.v.* intravenous, *MSC* mesenchymal stem cell, *Pdx-1* pancreatic and duodenal homeobox 1, *STZ* streptozotocin, *T*_*regs*_ regulatory T cells, *mMSC* murine mesenchymal stem cell, *BALB/c-MSC* Bagg Albino mesencymal stem cells

#### MSCs promote the survival and regeneration of existing β cells

Since MSCs are capable of modifying the tissue microenvironment, MSC infusions can promote the survival and regeneration of existing β cells, leading to increases in β-cell mass and restoration of normoglycaemia [21–24]. In fact, following intravenous injection of MSCs into diabetic mice, increases in insulin levels and reduced hyperglycaemia were observed. Similarly, a single treatment of umbilical cord-MSCs in humans provided lasting reversal of autoimmunity that allowed regeneration of islet β cells and improvement in glycaemic control [25–28]. However, the success of such interventions is closely related to the time from diagnosis. In many cases, people with long-standing T1D would possess very little to no remaining β cells, and consequently would unlikely be capable of regenerating sufficient quantities of de novo β cells to ameliorate their hyperglycaemia.

#### MSC modulation of autoimmunity

As previously mentioned, MSCs possess wide-ranging modulatory effects on immune cells, and therefore their use in abrogating autoimmune diseases has been well documented. The autoimmune nature of T1D unsurprisingly ignited interest in the use of MSCs as a potential cell therapy. Several pre-clinical diabetic animal studies showed that transplantation of MSCs results in glycaemic restoration as a consequence of suppressed T-cell proliferation and increased T regulatory cell (T_reg_) presence within pancreatic islets [14–18]. In a recently published clinical trial, MSCs injected through liver puncture successfully reduced the levels of islet-cell antibodies, glutamic acid decarboxylase, and insulin antibodies of two patients in 12 months, suggesting immune-modulated cell tolerance [29].

#### Pancreatic islet and MSC co-transplantation

Alternatively, MSCs recruit and increase the numbers of immunosuppressive host cells during co-transplantation of islets to promote graft survival. Specifically, following human islet transplantation in an advanced humanized mouse model, human bone marrow-derived MSCs (BMSC) increased quantities of T_regs_ and caused immune tolerance of the transplanted islets [20]. In fact, the co-transplantation of MSCs and pancreatic islets was able to achieve a state of normoglycaemia in diabetic rats likely via MSC trophic factor secretion which protected the transplanted islets [30]. In addition, MSCs in close contact with pancreatic islets began to express *Pdx-1* (pancreatic and duodenal homeobox-1, a fundamental transcription factor in β-cell development) and to differentiate into IPCs.

The collective results of these pre-clinical studies [14–18, 20, 30] demonstrate the success of MSCs as an immunomodulatory cell therapy for the treatment of T1D. Nevertheless, solutions are required to overcome the challenge of the loss of immunosuppressive characteristics over time and the requirement for high cell doses. In addition, the fact that animal studies have shown that MSCs derived from diabetic sources were unable to suppress immune responses in animals with diabetes raises concern over the utility of autologously sourced MSCs [31], potentially limiting the isolation of MSCs to allogeneic sources.

### MSCs as targets for the generation of IPCs

Ex vivo generation of IPCs is commonly performed via differentiation of precursor/stem cells using unique chemical regimens [6–10]. Alternatively, IPCs can be generated via viral-mediated gene transfer resulting in the transdifferentiation of lineage-committed cells [3–5]. Currently, encapsulation of IPCs is necessary to prevent recurrent autoimmune destruction or allorejection [32]. Although micro-encapsulation technology for the most part protects IPCs from CD4^+^ T cell-mediated destruction, these capsules are not impervious to cytokine-induced apoptosis. MSCs are an attractive target for generating IPCs due to their reported immune-modulatory properties. Potentially, IPCs generated from MSCs may retain some MSC-like qualities that could eliminate the need for encapsulation. Currently, there are two methods of generating IPCs from MSCs: chemically induced and viral-mediated transdifferentiation.

#### Chemically induced transdifferentiation

Deriving IPCs from stem cells has most commonly been performed by adapting in vitro transdifferentiation protocols to yield high numbers of fully functional IPCs [6, 7]. IPCs can be obtained from MSCs via the use of high-glucose culture medium [8, 9] or nicotinamide-enriched medium to induce transdifferentiation [10]. The resulting differentiated cells express insulin at both the mRNA and protein level, and ameliorate hyperglycaemia in STZ rats [10]. Similarly, BMSC isolated for expression of primitive stage-specific embryonic antigen-1 (SSEA-1) and a number of MSC markers such as CXC, octamer-binding transcription factor-4, and stem cell antigen-1 were induced to differentiate into IPCs in defined transdifferentiation medium [11]. These SSEA-1^+^ cells could further differentiate into islet-like clusters that stained positive with dithizone staining and were capable of secreting insulin in a glucose-responsive manner. MSCs derived from alternative tissue sources have also been subjected to similar chemically defined transdifferentiation protocols that resulted in the successful generation of IPCs [33, 34].

#### Viral-mediated transdifferentiation

Viral-mediated gene transfer has been investigated as a method to re-introduce normal copies of DNA into abnormal cells, or other cell types, as a means of treating genetic diseases for some time. Ideally, β-cell engineering would employ integrating viral vectors that provide sustained therapeutic gene expression over the life of the patient. To this end, viral-mediated transdifferentiation is an attractive method of obtaining surrogate β cells as they would be less likely to express identical autoantigens against which the primary autoimmune response was developed. Since transcription factors play a significant role in determining islet-cell fate during pancreatic embryogenesis, our laboratory and many others have investigated the direct transfer of β cell transcription factors and insulin as mediators of pancreatic transdifferentiation [3–5, 35].

Due to the success of gene transfer in generating IPCs from a number of cell types, and the requirement for encapsulation to translate basic research into the clinical setting, MSCs have become an attractive target for combinatorial gene and cell therapy. Consequently, several studies were performed investigating the ex vivo targeting of MSCs for viral-mediated transdifferentiation into IPCs. A study by Karnieli et al. utilised retrovirus for the transduction of BMSCs with the key pancreatic transcription factor *Pdx-1*, resulting in glucose-responsive production of insulin [12]. Furthermore, when these cells were transplanted under the renal capsule of STZ-diabetic SCID mice, they reduced blood glucose levels for a period of 6–8 weeks, after which abnormal glucose tolerance was observed. In fact, *Pdx-1* has been delivered to a number of tissue-specific MSCs, including BMSCs [13] and adipose-derived MSCs [36, 37] with varying success in generating glucose-responsive IPCs. Despite the early success with *Pdx-1*, some studies have shown that *Pdx-1* induced exocrine transdifferentiation, resulting in the development of hepatitis and an increased likelihood of autoimmune destruction [35]. Thus, alternative choices of transcription factors were assessed for their ability to induce pancreatic transdifferentiation without exocrine transdifferentiation. One study in particular found that *NeuroD1*-betacellulin delivery resulted in no hepatotoxicity, identifying *NeuroD1* as an ideal alternative for IPC generation [35]. These results were verified by our laboratory, where the transduction of the H4IIE rat liver cell line with rat *NeuroD1* and INS-FUR induced pancreatic transdifferentiation characterised by expression of β cell transcription factors, glucose-stimulated insulin secretion, and reversal of diabetes upon transplantation in STZ-diabetic mice [38].

Insulin transfer to MSCs has been performed less often, most likely due to the fact that ex vivo transfer of insulin alone is insufficient to induce pancreatic transdifferentiation, and most commonly results in constitutive insulin secretion. Xu et al. studied the retroviral transduction of BMSCs expressing the human insulin gene under the control of the cytomegalovirus (CMV) promoter and the ability of these transduced cells to restore normoglycaemia in STZ-diabetic mice [39]. The results showed that BMSCs successfully expressed insulin and were able to maintain normoglycaemia for at least 42 days. In addition, transduced BMSCs were able to evade autoimmune destruction that ordinarily targets pancreatic islets. However, similar efforts utilising adeno-associated virus (AAV) and retroviral vectors have resulted in constitutive insulin secretion as a consequence of the absence of glucose transporter 2 (GLUT2) and islet glucokinase gene expression. This feature would only develop following transdifferentiation rather than forced insulin expression. Thus, induction of pancreatic transdifferentiation for the production of IPCs should be mediated via transfer of specific pancreatic transcription factors, either alone or in combination, and in addition to the expression of insulin. The success of MSC-derived IPCs in pre-clinical animal models of diabetes is summarised in Table 1.

### From pre-clinical success to clinical failure and the way forward

Due to the success of MSC infusions and MSC-derived IPCs in pre-clinical animal models of diabetes, several human clinical trials have been undertaken or are currently in progress (Table 2). In translating pre-clinical studies to the human clinical setting, many of those conducting clinical trials have failed to consider the scientific basis of MSC function that makes their use in the pre-clinical setting so successful. Maintenance of MSC function for successful clinical trials is highly linked to choice of donor, preliminary ex vivo cell culture conditions, cryopreservation, and post-storage culture conditions, four caveats that have been overlooked and are a hallmark of the current clinical failure of MSCs for the treatment of T1D and other diseases.

**Table 2:** Summary of the major clinical trials utilising MSCs as a treatment for T1D

Trial number	Phase	Intervention	Outcomes	Fresh/frozen	Status	Reference
NCT01068951	N/A	Intravenous, autologous transplantation of MSCs (approximately 2 × 10^6^ cells/kg body weight)	Patients in the control arm showed losses in both C-peptide peak values and C-peptide when calculated as area under the curve during the first year. In MSC-treated patients, these responses were preserved or even increased. No side effects of MSC treatment were observed	Fresh	Completed 2014	[20]
NCT01374854	1/2	1 × 10^6^/kg UC-MSCs are infused through the pancreatic artery along with BM-MNCs by interventional therapy and another same dose of UC-MSCs administered 1 week post-intervention	C-peptide increased 105.7% in 20 of 21 responders versus 7.7% decrease in control subjects. HbA1C decreased 12.6% in treated versus 1.2% increase in control subjects. Daily insulin requirements decreased 29.2% in treated versus no change in control subjects.	Fresh	Completed 2012	[21]
NCT00703599	1/2	i.v. administration of autologous activated stromal vascular fraction derived from 100–120 ml lipoaspirates following mini-liposuction of abdominal adipose tissue	Not reported	Frozen	Estimated completion of 2009	[22]
NCT01219465	1/2	i.v transfusion of UC-MSCs (2 × 10^7^ cells/kg body weight)	No reported acute or chronic side effects in MSC-treated versus saline control. Both HbA1c and C-peptide in MSC-treated patients were significantly better than either pre-therapy values or saline control patients during the follow-up period	Fresh	Completed 2012	[23]
NCT01996228 NCT01350219	1/2	Human UC-MSCs within the Stem Cell Educator device	A single treatment provided lasting reversal of autoimmunity that allowed regeneration of islet β cells and improvement of metabolic control in subjects with long-standing T1D	Fresh	Recruiting	[24–27]
NCT02057211	2	Transfusion of autologous MSC versus sham MSC transfusion vs placebo control	N/A	Fresh	Recruiting	N/A
NCT01143168	1	Multiple transplantation of BM-MNC + UC-MSCs	Not reported	Frozen	Estimated completion of 2011	Cellonis Biotech Pty Ltd.
NCT00646724	1/2	Co-transplantation of islet allograft and MSC autograft	Not reported	Frozen	Estimated completion of 2012	N/A
NCT01322789	1/2	Four consecutive intravenous infusions, 1 week apart, followed by four consecutive infusions 1 month apart	Not reported	Frozen	Estimated completion of 2015	N/A
NCT01496339	1/2	1 × 10^6^/kg MenSCs are infused through the pancreatic artery or intravenously once a week in four consecutive therapies	Not reported	Frozen	Estimated completion of 2014	S-Evans Biosciences Pty Ltd.
NCT02644759	1/2	Transplantation of autologous CD34^+^/CD133^+^ cells into the pancreatic artery and capillaries via interventional radiology techniques. Immunomodulation by incubation of autologous UC-MSCs for 3–6 h, and return of autologous WBCs back via intravenous injection	N/A	Fresh	Ongoing, not recruiting	Stem Cells Arabia
NCT00690066	2	Intravenous infusion of ex vivo cultured adult human MSCs vs placebo intravenous infusion of excipients of PROCHYMAL®	Not reported	Frozen	Completed 2014	Osiris/Mesoblast International Sarl
NCT01157403	2/3	Intravenous autologous transplantation of BMSC (approximately 2.5 × 10^6^ cells/kg body weight)	Not reported	Frozen	Estimated completion of 2014	N/A

Clinical trial data was acquired from www.clinicaltrails.gov using the search terms “mesenchymal stem cells” and “type 1 diabetes”

*BM-MNC* bone marrow mononuclear cell, *BMSC* bone marrow-derived mesenchymal stem cell, *CD* cluster of differentiation, *HbA1C* glycosylated haemoglobin, *i.v.* intravenous, *MenSC* menstrual blood mesenchymal stem cell, *MSC* mesenchymal stem cell, *N/A* not available, *T1D* type 1 diabetes, *UC-MSC* umbilical cord mesenchymal stem cell, *WBC* white blood cell

#### MSC donor profiling and post-harvest expansion

In many clinical trials, MSCs are harvested from a single donor, culture expanded, and cryopreserved prior to administration to a trial participant. Donor variability is a concern that requires careful assessment prior to harvesting MSCs for clinical trials, as phenotype, age, and gender can affect MSC function and growth characteristics [40]. Consequently, the choice of a suitable donor is critical, as MSCs with a dampened immunomodulatory profile are less likely to perform as well in vivo as MSCs with a robust immunomodulatory profile. As such, profiling of multiple donors for MSC immunomodulatory function should be included as a necessary control step prior to the utilisation of allogeneic MSC treatments.

Compounding single donor harvesting is the fact that MSCs constitute a small proportion of the cellular fraction of the tissue from which they are sourced. Thus, culture expansion is required to generate clinically sufficient quantities of MSCs for transplantation, a process that results in phenotypic changes such as a decrease in proliferative capacity and self-renewal. In addition, doubts concerning the clinical application of ex vivo cultured MSCs for immunomodulatory therapies have been raised due to their apparent loss of immunomodulatory properties with continued subculture (in particular the downregulation of chemokine receptors and reduction in secretion of soluble factors). This is highlighted by the Prochymal® study (Table 2) which pursued the off-the-shelf “one harvest, multiple therapies” concept, and ultimately failed in the clinic due to the extensive ex vivo culture required. The major contributing factor to the success or failure in pre-clinical vs clinical trials of MSCs is that pre-clinical studies utilise freshly harvested MSCs from either single or multiple animal donors followed by immediate transplantation into the animals to be treated. Minor cell expansions are acceptable; however ongoing expansion for upscaling of cell quantities ultimately detrimentally affects MSCs in the clinical setting. Reconstituting the downregulation of MSC chemokine receptors and secreted factors via gene transfer to prolong MSC immunomodulatory properties for clinical therapies is a solution that could prove beneficial, especially with regards to T1D which requires a long-term therapeutic solution. However, multiple gene transfer of exogenous genes represents a logistical challenge that would render such a therapy challenging to translate into the clinic.

#### Post-cryopreservation culture and administration

Naturally, storage of therapeutic cells is a crucial process in conducting clinical trials. With trial participants required to travel long distances to receive treatment, the need to preserve stocks of therapeutic cells in the interim is imperative. Cryopreservation is the most popular form of cell preservation, and in the clinical setting is a practical form of bio-banking. However, several studies have shown that cryopreserved MSCs have an impaired immunosuppressive profile in comparison to fresh MSCs, explaining the failure of many clinical trials utilising cryopreserved, culture-expanded MSCs [41, 42]. This feature is characterised by increases in expression of heat shock proteins, a lack of interferon (IFN)-γ-mediated indoleamine 2,3-deoxygenase (IDO) production and a reduced immunosuppressive effect on T-cell populations. Interestingly, post-cryopreservation culture for 24 h reverses these effects [41]. As such, successful clinical use of MSCs for the treatment of T1D (and many other diseases) requires careful trial organisation, focusing on the appropriate and responsible use of MSCs. In future, regulation of critical processes such as donor choice, minimal post-harvest expansion, and post-freeze MSC culture could result in more successful clinical trials that fulfil the promise of pre-clinical studies and that of the exciting prospect of MSCs as a cellular therapy for T1D.

### CRISPR: a novel strategy to improve MSC therapy for T1D

In recent times, a novel gene-editing technology that has caught the interest of scientists worldwide is the class of RNA-guided endonucleases known as CRISPR-associated protein 9 (Cas9) from the microbial adaptive immune system known as CRISPR [43, 44]. Using short guide (sg)RNAs, the Cas9 endonuclease can be guided to any genomic location, inducing double strand breaks allowing for non-homologous end joining or homologous recombination of genomes at specific locations. By inactivating the catalytic activity of the Cas9 endonuclease, a nuclease-deficient Cas9 (dCas9) can be engineered. In fact, dCas9 fused to transactivation domains such as VP64 or p300 can subsequently be guided using target-specific sgRNAs to the upstream promoter region of endogenous genes, thereby upregulating gene expression [43, 44]. In addition, concerns regarding off-target effects are not as pronounced by comparison to traditional CRISPR applications due to the promoter-specific design of sgRNAs that possess less likelihood of unwanted activator recruitment to promoter regions of non-target genes by chance.

Early studies showed that single sgRNAs targeted to the promoter region of target genes activate negligible levels of endogenous gene expression. However, multiplexing with three or more sgRNAs results in synergistic activation of target genes with significant increases in gene expression. More importantly, sgRNA multiplexing can be designed for multiple gene activation [45], thereby eliminating the need to deliver multiple transcription factor cDNAs for robust gene expression to induce differentiation. Improvement in dCas9-transcriptional activator fusions have led to novel gene activators with enhanced activation capabilities compared to early dCas9-VP64 activators. Fusions of dCas9 to multiple co-activators such as the VPR domain (consisting of VP64, p65, and Rta), or specifically engineered dCas9 proteins that utilize modified sgRNAs in conjunction with a synergistic activator complex have been shown to induce robust gene activation [46]. In fact, several recent studies have demonstrated CRISPR-mediated activation of endogenous gene expression for controlled differentiation into diverse cell types [47–49].

**Fig. 1:**
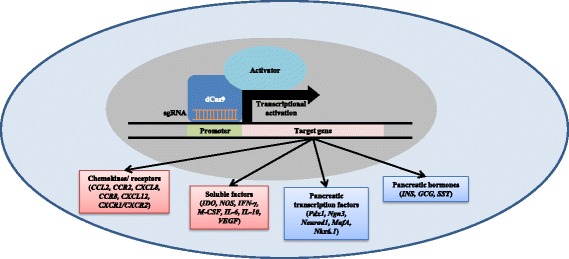
CRISPR-mediated generation of IPC and enhanced MSC-derived immunotherapies. Expression of the nuclease-deficient dCas9 fused to a transcriptional activator in MSCs facilitates the activation of endogenous gene expression. To drive the differentiation of MSCs into IPCs (*blue boxes*), sgRNAs targeting the promoter of target genes such as *Pdx-1*, *NeuroD1*, *MafA*, etc., are delivered to MSCs expressing the dCas9-transcription activator fusion. Additional sgRNAs targeting the promoter of genes involved in MSC immunomodulation (*red boxes*) are delivered in combination to maintain the immunomodulatory phenotype of MSCs through multiple cell expansions for the development of therapeutic doses of cell therapy. *CCL* chemokine ligand, *CXCR* chemokine receptor, *dCas9* nuclease-deficient CRISPR-associated protein 9, *GCG* glucagon, *IDO* indoleamine 2,3-deoxygenase, *IFN* interferon, *IL* interleukin, *INS* insulin, *MafA* v-maf musculoaponeurotic fibrosarcoma A, *M-CSF* macrophage colony stimulating factor, *Neurod1* neuronal differentiation 1, *Ngn3* neurogenin 3, *Nkx6.1* NK6 homeobox 1, *NOS* nitric oxide synthase, *Pdx1* pancreatic and duodenal homeobox 1, *sgRNA* short guide RNA, *SST* somatostatin, *VEGF* vascular endothelial growth factor

**Fig. 2:**
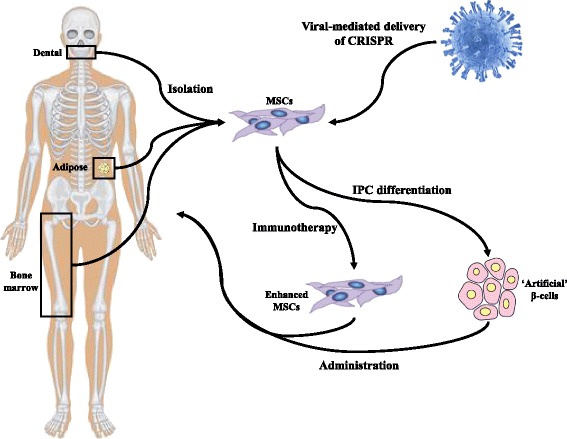
Clinical application of CRISPR for the treatment of T1D MSCs are isolated from a variety of adult tissue sources including bone marrow, adipose tissue, and dental pulp for ex vivo culture. MSCs are subsequently gene modified with the nuclease-deficient dCas9-transcriptional activator CRISPR complex and specific sgRNA for the desired purpose of either generating enhanced MSCs that maintain their immunomodulatory properties as an immunotherapy, or for the generation of IPCs. The generated therapy is subsequently administered to a trial participant either systemically (immunotherapy) or subcutaneously (IPCs) in an area conducive to vascularisation of the transplanted IPCs. *CRISPR* clustered regularly interspaced short palindromic repeat, *MSC* mesenchymal stem cell, *IPC* insulin-producing cell

By utilizing dCas9 activators and multiple sgRNAs to target the endogenous activation of pancreatic transcription factors and/or MSC chemokine receptors in MSCs, it may be possible to direct the differentiation of MSCs into surrogate IPCs capable of maintaining their immunomodulatory properties through ex vivo expansion and transplantation (Fig. 1). To this end, one study in particular has demonstrated success in activating endogenous human insulin transcription utilizing the dCas9-VP160 fusion and multiple insulin promoter targeting sgRNAs in HEK293T, Hela, and human fibroblasts [50]. The success of this study supports the proposed novel application of activating transcription of endogenous genes involved in pancreatic development such as *Pdx-1*, *Neurod1*, *MafA*, etc. When combined with the targeted activation and maintenance of genes involved in MSC immunomodulation such as chemokine receptors and soluble factor production, the likelihood of developing successful MSC-derived therapies for T1D becomes a realistic outcome (Fig. 2).

## Conclusion

Both gene therapy and stem cell therapy have proven to be successful avenues for the development of a treatment for T1D. In vitro and in vivo studies have shown that both systems have the potential to be developed further, either individually or in combination. Although MSCs have been shown to be successful in treating T1D by modulating the pancreatic islet microenvironment and immune responses in preclinical and clinical studies, a sustained therapeutic effect is yet to be observed. Similarly, the use of IPCs generated from autologous MSCs to reverse T1D is limited by several challenges such as recurrent autoimmunity, obtaining sufficient numbers of IPCs for transplantation, and loss of immunomodulatory properties. Ultimately, the use of MSC-derived therapies for the treatment of T1D requires appropriate manufacturing conditions that do not impinge on the function of MSCs prior to administration. Clinical success will require perseverance and realistic translation of pre-clinical methodology, without pushing the applicable limits of these therapies.

## Abbreviations

AAV: Adeno-associated virus
BMSC: Bone marrow-derived mesenchymal stem cell
Cas9: CRISPR-associated protein 9
CD: Cluster of differentiation
CRISPR: Clustered regularly interspaced short palindromic repeat
GLUT2: Glucose transporter 2
IDO: indoleamine 2,3-deoxygenase
IFN: Interferon
INS-FUR: Furin cleavable human insulin
IPC: Insulin-producing cell
*MafA*: V-Maf avian musculoaponeurotic fibrosarcoma oncogene homolog A
MSC: Mesenchymal stem cell
*NeuroD1*: Neurogenic differentiation 1
NOD: Non-obese diabetic
*Pdx-1*: Pancreatic and duodenal homeobox-1
sgRNA: short guide RNA
SSEA-1: Stage-specific embryonic antigen-1
STZ: Streptozotocin
T1D: Type 1 diabetes
T_reg_: T regulatory cell
